# Characterization of the complete chloroplast genome of a common Chinese medicinal herb, *Eriocaulon buergerianum* (Eriocaulaceae: *Eriocaulon*)

**DOI:** 10.1080/23802359.2019.1673252

**Published:** 2019-10-09

**Authors:** Mingliang Shan, Tianyi Cao, Wenhao Xing, Chengwei Guo

**Affiliations:** a Shandong University of Traditional Chinese Medicine, Jinan, PR China;; b Integrated Traditional Chinese and Western Medicine Hospital, Hangzhou, PR China;; c Affiliated Hospital, Shandong University of TCM, Jinan, PR China

**Keywords:** *Eriocaulon buergerianum*, Eriocaulaceae, Chinese medicinal herb, chloroplast genome, evolutionary analysis

## Abstract

*Eriocaulon buergerianum* is a common Chinese medicinal herb and belongs to the family Eriocaulaceae genus Eriocaulon the annual herbs. In this study, the complete chloroplast genome of *E. buergerianum* was assembled and reported. The complete chloroplast genome of *E. buergerianum* is 157,016 bp in length as the circular, which harbours a large single-copy region (LSC) of 81,534 bp, a small single-copy region (SSC) of 17,114 bp, and two inverted-repeat regions (IRs) of 26,393 bp each one. The overall nucleotide content of the chloroplast genome: A of 31.8%, T of 32.4%, C of 18.2% G of 17.6%, and 35.8% GC content. The chloroplast genome of *E. buergerianum* contains 133 genes, which includes 88 protein-coding genes (PCGs), 37 transfer RNA (tRNAs), and 8 ribosome RNA (rRNAs). The evolutionary analysis used neighbour-joining (NJ) method and the result showed that *E. buergerianum* was closely related to *Eriocaulon sexangulare* in the family Eriocaulaceae. This study will be helpful for genome data and genomic resources of the family Eriocaulaceae for further.

*Eriocaulon buergerianum* is a common Chinese medicinal herb and belongs to the family Eriocaulaceae genus Eriocaulon, which also is the annual herb. Plant whole of *E. buergerianum* is named Gu-Jing-Cao in Chinese (Ho and Chen 2002). Thirteen genera is in the family Eriocaulaceae, which the genus Eriocaulon contains around 435 species distributed throughout the world (Ho and Chen 2002). Previous phytochemical research revealed *E. buergerianum* of the genus Eriocaulon led to the identification of flavonoids, naphthopyranones and γ-tocopheryl acetate (Santos et al. 2005). *E. buergerianum* is frequently used as anti-inflammatory and antimicrobial medicine in China. Now, we do not have knowledge about the chloroplast genome and other genome information of the *E. buergerianum*. So, in this study, the complete chloroplast genome of *E. buergerianum* was assembled, annotated, and reported, which can be valuable for genome data and genomic resources for further, also can be important for evolutionary analysis and utilization of the Eriocaulaceae family.

Plant whole of *E. buergerianum* was collected from Shandong University of Traditional Chinese Medicine in Jinan, Shandong, China (36.65°N, 117.05°E). The total genomic DNA of *E. buergerianum* was extracted from the whole plant using the modified CTAB method and stored in Shandong University of Traditional Chinese Medicine (No.SUTCM-01). The genomic DNA was purified and fragmented using the NEB Next Ultra^TM^ II DNA Library Prep Kit (NEB, BJ, and CN), which was sequenced. FastQC version 0.11.8 (Andrews 2015) was used to perform and remove low-quality reads and adapters for quality control. The chloroplast genome of *E. buergerianum* was assembled and annotated using the MitoZ (Meng et al. 2019). OrganellarGenomeDRAW version 1.3.1 (Greiner et al. 2019) was used to draw the physical map of the chloroplast genome of *E. buergerianum*.

The complete chloroplast genome of *E. buergerianum* is 151,434 base pairs (bp) in length which is the circular with the overall nucleotide content of the chloroplast genome: 31.8% A (Adenine), 32.4% T (Thymine), 18.2% C (Cytosine), 17.6% G (Guanine), and 35.8% GC content. It harbours a characteristic quadripartite structure with a large single-copy region (LSC) of 81,534 bp, a small single-copy region (SSC) of 17,114 bp, and two inverted repeat regions (IRs) of 26,393 bp. The chloroplast genome of *E. buergerianum* contains 133 genes, which includes 88 protein-coding genes (PCGs), 37 transfer RNA genes (tRNAs), and 8 ribosomal RNA genes (rRNAs). And 20 genes were found duplicated in IR regions at each one, which included 8 PCG genes species (*rps19, rpl2, rpl23, ycf2, ndhB, rps7, rps12*, and *ycf1*), 8 tRNA genes species (*trnH-GUG, trnI-CAU, trnL-CAA, trnV-GAC, trnI-GAU, trnA-UGC, trnR-ACG*, and *trnN-GUU*), and 4 rRNA genes species (*rrn16, rrn23, rrn4.5*, and *rrn5*). The complete chloroplast genome of *E. buergerianum* has submitted to the GenBank that NCBI accession No.MH9677841.

To further investigate *E. buergerianum* evolutionary and phylogenetic position, the neighbour-joining (NJ) tree was constructed based on the chloroplast genome sequences of 10 other species using MEGA X (Kumar et al. 2018). NJ tree analysis used NJ method and performed using 2000 bootstrap values replicate at each node. All of the nodes were inferred with strong support by the NJ methods. The final NJ evolutionary tree was edited using the iTOL version 4.0 online web (https://itol.embl.de/) (Letunic and Bork 2016). The evolutionary tree analysis result showed that *E. buergerianum* was closely related to *Eriocaulon sexangulare* (MK193813.1) in the family Eriocaulaceae (Figure 1). In a word, the complete chloroplast genome of *E. buergerianum* is very important for Chinese medicinal herb research value and clinical drug development for further.

**Figure 1.**
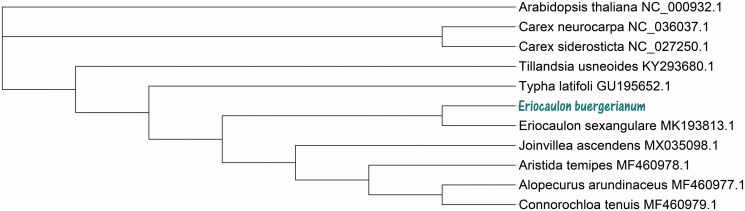
Phylogenetic relationships of 11 species chloroplast genome sequences based on the neighbour-joining (NJ) method analysis using 2000 bootstrap replicates. Bootstrap support is indicated for each branch.
